# Regulatory effects of IRF4 on immune cells in the tumor microenvironment

**DOI:** 10.3389/fimmu.2023.1086803

**Published:** 2023-02-06

**Authors:** Jing Lu, Taotao Liang, Ping Li, Qingsong Yin

**Affiliations:** ^1^ Department of Hematology, The Affiliated Cancer Hospital of Zhengzhou University and Henan Cancer Hospital, Zhengzhou, Henan, China; ^2^ Department of Hematology, The University of Texas MD Anderson Cancer Center, Houston, TX, United States

**Keywords:** IRF4, tumor microenvironment, immunosuppressive cells, T cell exhaustion, immunoregulation

## Abstract

The tumor microenvironment (TME) is implicated in tumorigenesis, chemoresistance, immunotherapy failure and tumor recurrence. Multiple immunosuppressive cells and soluble secreted cytokines together drive and accelerate TME disorders, T cell immunodeficiency and tumor growth. Thus, it is essential to comprehensively understand the TME status, immune cells involved and key transcriptional factors, and extend this knowledge to therapies that target dysfunctional T cells in the TME. Interferon regulatory factor 4 (IRF4) is a unique IRF family member that is not regulated by interferons, instead, is mainly induced upon T-cell receptor signaling, Toll-like receptors and tumor necrosis factor receptors. IRF4 is largely restricted to immune cells and plays critical roles in the differentiation and function of effector cells and immunosuppressive cells, particularly during clonal expansion and the effector function of T cells. However, in a specific biological context, it is also involved in the transcriptional process of T cell exhaustion with its binding partners. Given the multiple effects of IRF4 on immune cells, especially T cells, manipulating IRF4 may be an important therapeutic target for reversing T cell exhaustion and TME disorders, thus promoting anti-tumor immunity. This study reviews the regulatory effects of IRF4 on various immune cells in the TME, and reveals its potential mechanisms, providing a novel direction for clinical immune intervention.

## Introduction

The occurrence and development of tumors highly depend on the surrounding matrix environment, called the tumor microenvironment (TME). The oncogene proteins expressed by tumor cells stimulate and induce the abnormal activation of effector T cells (1, 2). Multiple soluble tumor-derived products, such as the chemokines CCL2, CCL5 and the cytokines IL10 and TGFβ, etc., recruit tumor-associated macrophages (TAMs) (3–6) and myeloid-derived suppressor cells (MDSCs) (7) into the TME, and lead to the impairment of differentiation, maturation and function of dendritic cells (DCs) (8, 9). These factors in turn jointly aggravates TME disorders, inhibits the anti-tumor immunity of effector T cells, and induces T cell exhaustion and the development of regulatory T (Treg) cells (2). As a result, apart from genetic deficiencies, the immunosuppressive TME is considered to be involved in tumorigenesis (10), chemoresistance, immunotherapy failure and even tumor recurrence (2, 6).

Given this reliance on the TME, there is an opportunity for anti-tumor immunotherapies that work by targeting TME components and their signaling pathways (11, 12). Although tremendous progress has been made in the past few years, including immune checkpoint inhibitors (13), bispecific antibodies (14) and chimeric antigen receptor (CAR) T cells (15), many studies focusing on elements of the TME have failed to show promising efficacy in patients, particularly with sustainable efficacy (16–18). Therefore, the development of new immunotherapies may also require consideration of the key transcription regulatory factors involved in multiple components and processes in the TME.

**Figure 1:**
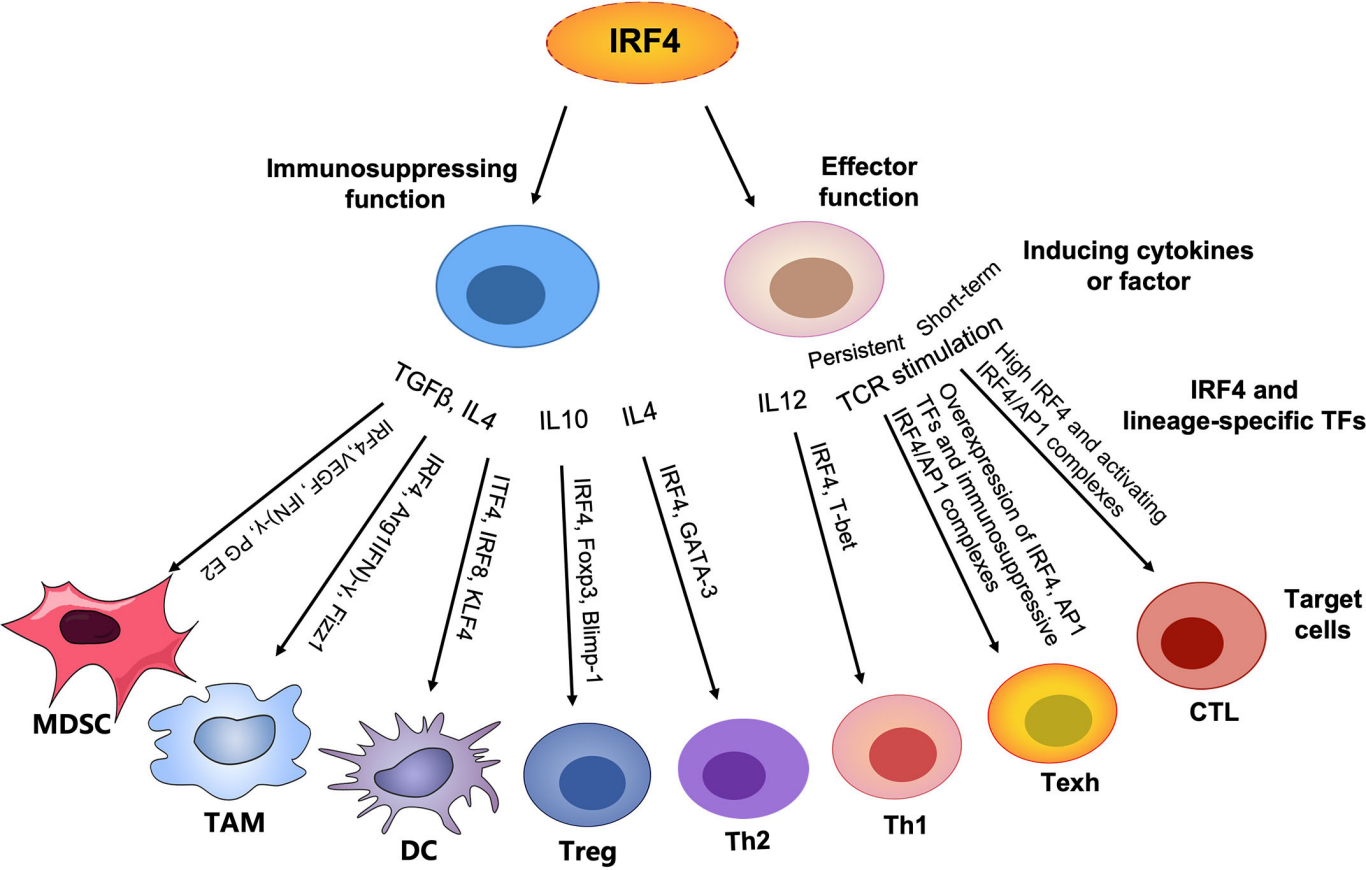
Graphical abstract. In contrast to lineage-specific TFs, IRF4 plays an important role in T cell differentiation and function by regulating the expression of corresponding transcription factors (TFs) to control the generation of other lineages, particularly the differentiation and proliferation of effector T cells, promoting anti-tumor immunity. However, persistently high expression of IRF4 and AP1 family members leads to overabundance of IRF4/AP1 complexes to drive T cell exhaustion. In addition, IRF4 plays an important role in the development and function of a series of immunosuppressive cells, such as MDSCs, TAMs, immature DCs, Treg cells and Th2 cells, maintaining immune homeostasis and in parallel establishing an immunosuppressive TME and inhibiting anti-tumor immunity.

Interferon regulatory factor 4 (IRF4) is a member of the interferon regulatory factor (IRF) family, and its unique characteristics and the importance in multiple biological processes have been highlighted by oncology and immunology. It first serves as an oncogene or a tumor suppressor in multiple types of lymphoid neoplasms (19–21). In addition, intriguingly, accumulating studies have demonstrated that IRF4 is a central determinant of differentiation, activation and effector function for various immune cells (22, 23). IRF4 is essential for the sustained differentiation and proliferation of CD8+ cytotoxic T cells (CTLs) and T helper 1 (Th1) cells, promoting anti-tumor immunity. In parallel, IRF4 is also involved in T cell exhaustion in specific biological contexts (24, 25). In contrast, it plays an important role in the differentiation and function of various immunosuppressive cells, such as Th2 cells, Treg cells, TAMs and MDSCs, establishing an immunosuppressive TME to inhibit anti-tumor immunity and favor the immune escape and survival of tumor cells (3–5, 7) (**Figure 1**). Thus, an in-depth understanding of the effects and potential mechanisms of IRF4 in a variety of immune cells and a disordered TME may provide new directions for clinical immune intervention.

## Structure and function of IRF4

The IRF family consists of nine members (IRF1-IRF9) in mammals that play important roles in regulating innate and adaptive immune responses. Unlike other IRFs, IRF4 is a unique family member that is not regulated by interferons (IFNs) (22), instead, is mainly induced upon T-cell receptor (TCR) signaling, Toll-like receptors (TLRs; such as TLR4 and TLR9) and tumor necrosis factor receptors. The expression of IRF4 is restricted to immune cells, including T and B cells, macrophages and DCs (19, 22). In naïve T cells, IRF4 is expressed at low levels (23); however, following TCR signaling it is immediately induced and mediates critical immune responses by interacting with upstream signaling pathways, such as the TCR signaling, and its diverse binding partners (26).

**Figure 2:**
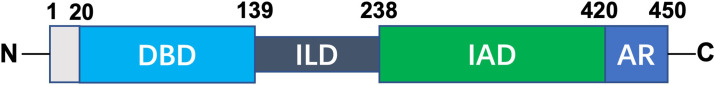
Schematic diagram of IRF4 structure. IRF4 consists of three structural domains: a highly conserved N-terminal DNA-binding domain (DBD), a variable C-terminal IRF association domain (IAD) and an intermediate linker domain (ILD). The DBD is characterized by five conserved tryptophans enabling it to form a helix–loop–helix motif that facilitates DNA binding. IAD is a protein–protein interaction domain that mediates the interaction of IRF4 with itself or multiple distinct transcription factors. IAD also contains a C-terminal auto-inhibitory region (AR) which physically interacts with DBD and results in low DNA binding affinity.

IRF4 is composed of three structural domains: a variable C-terminal functional regulatory domain, a highly conserved N-terminal DNA-binding domain and an intermediate compact linker domain (22, 27, 28) (**Figure 2**). IRF4 interacts with numerous DNA-binding domains to play corresponding functions as a homodimer or heterodimer (29). IRF4 binds to interferon-stimulated response elements (ISREs) to regulate the activation of interferon-stimulated genes (ISGs) as a homodimer. However, the formation of heterodimeric complexes containing IRF4 depends largely on the target cell type. For instance, IRF4 engages activator protein 1 (AP1)-IRF composite elements (AICE) as a heterodimer mainly in T cells, germinal center B cells and plasma cells (23, 28). Whereas the binding of IRF4 with erythroblast transformation (ET)-specific transcription factors (TFs) is largely restricted to B cells and DCs. Of note, the binding of IRF4 to AICE requires AP1 family TFs, including basic leucine zipper transcription factor ATF-like (BATF), BATF3 and Jun family members, such as JunB, c-Jun, for high-affinity interaction (23, 30–33). These TFs form ternary complexes through physical interaction to coordinately regulate the differentiation and function of T cells, as well as T cell exhaustion, in a special microenvironment (24, 33–35).

Collectively, IRF4 can signal to regulate diverse transcriptional programs through complexes containing ET or AP1 TF motifs in different cell types depending on the corresponding cellular context, particularly T cell exhaustion in the TME, thus suggesting new directions for improving anti-tumor immunity by modulating IRF4-dependent transcription.

## Roles of IRF4 in the differentiation and function OF CD4+ T cells

According to different functions, CD4+ T cells can be divided into CD4+ effector T cells, including Th1, Th2 and Th17 cells, which predominantly promote the immune response, T follicular helper cells (Tfh), which orchestrate antibody responses (26), and Treg cells, which are characterized by their inhibition of the immune response and maintenance of immune tolerance (26, 36, 37). In contrast to lineage-specific TFs (e.g., T-bet for Th1, GATA3 for Th2, RORγt for Th17, B-cell lymphoma 6 (Bcl6) for Tfh and Foxp3 for Treg), TCR signaling-induced IRF4 plays an important role in Th cell differentiation and function by regulating the expression of corresponding TFs to control the generation of other lineages, thus determining the fate of Th cells (23, 26, 29, 38).

### IRF4 determines the fate of Th1, Th2, Th17 and Tfh

Th cell differentiation is regulated by the coordinated functions of distinct cytokines and transcription factors. A recent study has demonstrated that increased IRF4 promotes the differentiation of CD4+ CD25^low^ Teff cells, including Th1, Th2 and Th17 cells, at the expense of Tfh cells (26). In fact, the development and differentiation of Tfh cells only needs an appropriate amount IRF4 in addition to specific TFs, including Bcl-6 and signal transducer and activator of transcription 3 (STAT3) (26, 39). B-lymphocyte-induced maturation protein 1 (Blimp1) is a critical antagonist for Tfh cell differentiation, but it is an important TF for other Th cells, including Th1, Th2, Th17 and Treg cells (40). It has been found that IRF4 cooperates with STAT3 to activate Blimp1 (41), and lack of IRF4 in CD4+ T cells reduces binding to STAT3, resulting in Tfh deficiency (41, 42).

Increasing studies have shown that IRF4 regulates Th17 cell development (43–45). IRF4 knockout decreases the expression of RORγt, a specific TF in Th17 cells (45, 46), which leads to a decrease in Th17 counts, in line with a reduction in serum IL17 and IL21 (47). Likewise, IRF4 deficiency also results in the impairment of Th2 cell differentiation and function by reducing GATA3 and IL4, as well as growth factor independence 1 (Gfi1), a transcriptional repressor required by Th2 cells (48, 49), instead, can promote the T-bet expression and skew toward Th1 cells (48), suggesting that IRF4 plays a pivotal role in the development of Th2 cells rather than Th1 cells. Additionally, IRF4 deficiency inevitably impairs the development of Th2 cells (49). Collectively, IRF4 regulates the differentiation and function of diverse Th subsets that mainly depend on its expression level as well as lineage-specific TFs (26).

### IRF4 favors the development and suppressive activity of Tregs

Treg cells are indispensable for maintaining immune tolerance (37, 50); nevertheless, they also impair anti-tumor capability and promote tumor growth, particularly tumor-infiltrating Treg cells (51). Foxp3 is a lineage-defining TF for Tregs and the key regulator of its development and function (52, 53). IRF4, which acts downstream of Foxp3, can physically and functionally interact with Foxp3 and cooperate with BATF3 to regulate Foxp3 expression (54, 55), which instructs effector Treg cell differentiation and immune suppression (56). Moreover, Blimp1 is a target of Foxp3 in Treg cells, and it is directly induced by IRF4 (57, 58). Accordingly, lack of IRF4 in Treg cells suppresses Blimp1 expression, and more intriguingly, leads to decreases in multiple Treg-related molecules, such as inducible T cell costimulatory (ICOS), IL10 and IL1 receptor 11 (IL1RL1), confirming that IRF4 cooperates with Blimp1 to regulate the differentiation and function of Treg cells (56, 58).

Additionally, compared with IRF4-deficient Treg cells, IRF4+ Treg cells overexpress BATF, IKAROS family zinc finger 2 (IKZF2), Ki67, ICOS and inhibitory molecules, such as programmed cell death protein 1 (PD1) and T cell immunoreceptor with immunoglobulin and ITIM domain (TIGIT) (38), exhibiting a highly activated phenotype and strong inhibitory effects in several tumors (59–61). In particular, an increase in intratumoral IRF4+ Treg cells with superior suppressive activity was significantly correlated with early tumor recurrence and poor disease-free survival (DFS) and overall survival (OS) (38). Accordingly, inhibition of IRF4 severely impaired the development and function of Treg cells at the tumor-infiltrating sites and significantly repressed tumor growth in a mouse model (38, 51). Collectively, growing evidence implicates IRF4 plays a central role in the differentiation and immunosuppressive activity of Treg cells in the TME, and IRF4+ Treg cells definitely inhibit anti-tumor immunity. Therefore, specifically targeting IRF4 in Treg cells may reverse the tumor microenvironment from immunosuppression to immune activation against tumor cells, which may become an effective anti-tumor therapeutic strategy.

## Effect of IRF4 on the differentiation and function of CD8+ T cells

CD8+ T cells play critical roles in adaptive immunity. Antigen stimulation drives naïve CD8+ T cells to rapidly undergo a step-by-step process of early activation, clonal expansion and differentiation (62–65). In addition to early activation, IRF4 participates in the entire process of differentiation and function of effector CD8+ T cells (66, 67). Intriguingly, the amount and duration of IRF4 expression determine the fate of CD8+ T cells, which are differentiated into CD8+ effector T cells or exhausted T cells (24, 67–69).

### High IRF4 promotes the expansion and sustained differentiation of CD8+ T cells

**Figure 3:**
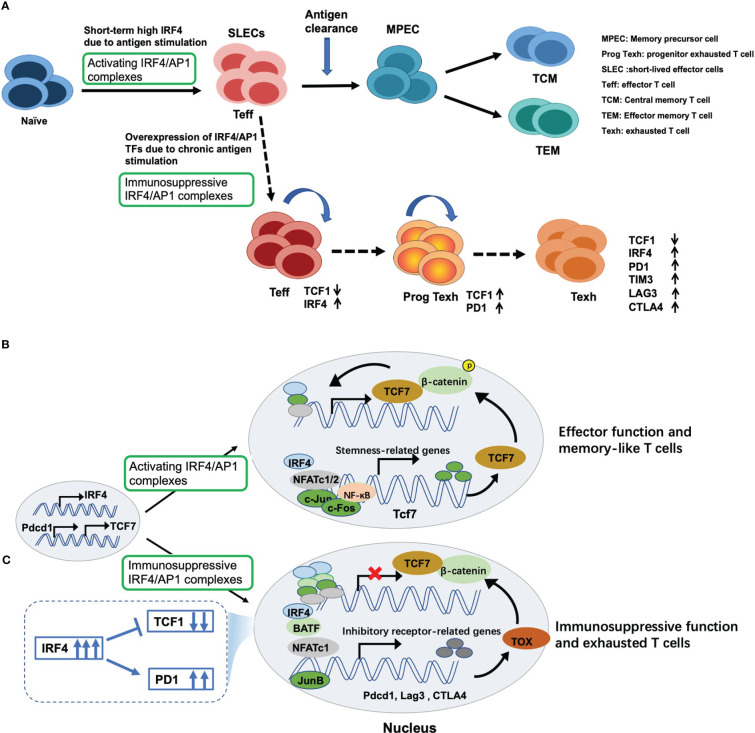
Dual regulatory effects of IRF4 on T cell immunity and underlying mechanisms. **(A)** Antigen stimulation drives and induces the expression of IRF4, which contributes to maintaining the expansion and sustained differentiation of effector CD8+ T cells. However, sustained overexpression of IRF4 due to chronic antigen stimulation drives CD8+ T cell exhaustion. Mechanically, **(B)** once antigen stimulation, IRF4 is induced and combined with its binding partners to form activating IRF4/AP1 complexes, thus inducing the production of effector cytokines and exerting cytotoxic activity. Once antigen clearance, the expression of IRF4 decreases, followed by an increase in expression of stemness-like gene TCF7 expression, thus producing TCF1+ memory-like T cells; **(C)** however, persistent overexpression of IRF4 and AP1 family members, such as BATF, BATF3 and JunB, leads to the formation of immunosuppressive IRF4/AP1 complexes, which opens multiple exhaustion-related chromatin regions, promoting the expression of inhibitory receptors and NR4A and TOX family members, which inhibits TCF7 expression and eventually drives CD8+ T cell exhaustion.

Following antigen stimulation, naïve CD8+ T cells are differentiated into a large number of antigen-specific short-lived effector cells (SLECs) (62, 63), exerting cytotoxic activity (**Figure 3A**). Mechanically, antigen stimulation drives the expression of TCR responsive factor IRF4 (68). Next, IRF4 combined with AP1 family TFs form an activating IRF4/AP1 complex, which integrates TCR and costimulatory signals to induce the production of a series of effector cytokines. After antigen clearance, the expression of IRF4 decreased, followed by an increase in expression of stemness-like gene T cell factor 7 (*Tcf7*; encoding TCF1) (**Figure 3B**), and further producing memory precursor cells (MPECs) and TCF1+ memory-like T cells to rapidly function in the secondary response (64, 65) (**Figure 3A**).

The intensity of TCR signaling regulates the expression of IRF4 (66, 70). High levels of IRF4 in CD8+ T cells contribute to the clonal expansion of SLECs, which are critical for maintaining effective anti-tumor immunity (71) and acute pathogen control (64). Interestingly, ectopic expression of IRF4 remarkably enhances the clonal expansion and effector cytokine production of T cells induced by low-intensity TCR signaling (69). Conversely, selective knockout of IRF4 in peripheral CD8+ T cells leads to progressive loss of the effector function of CD8+ T cells (72–74). The RNA-binding protein Roquin1, a key target upstream of IRF4, can effectively inhibit the expansion of CD8+ T cells (75). Accordingly, lack of Roquin1 can significantly promote the proliferation of CD8+ T cells by upregulating IRF4 (71). However, if IRF4 is also deficient, the expansion-promoting effects caused by Roquin1 deficiency is completely abolished (71). Therefore, the Roquin-IRF4 axis may also serve as a potential target for enhancing anti-tumor immunity.

IRF4 also converts TCR affinity into appropriate transcriptional programs, linking metabolic function to T cell clone expansion and effector differentiation (76) by regulating the expression of key molecules required for aerobic glycolysis on effector T cells, including hypoxia inducible factor1 α (HIF1α) and forkhead box protein o1 (Foxo1) (77). Compared with weak or low-affinity TCR stimulation, strong or high-affinity TCR stimulation contributes to increased glucose uptake in an IRF4-dependent manner (78). Taken together, IRF4 regulates the expansion and differentiation of effector CD8+ T cells by translating the TCR signal and converting it to metabolic function.

### IRF4 maintains the effector function of CD8+ memory T cells

Not surprisingly, similar to initial antigen stimulation, IRF4 overexpression significantly induces an increase in the cytotoxicity of memory CD8+ T cells (32, 68, 79). By contrast, IRF4 deficiency may cause memory CD8+ T cells to produce but not proliferate (68), which results in impairment of the effector function (32, 72, 79). So far, at least three types of memory CD8+ T cells have been defined: central memory T (T_CM_) cells, effector memory T (T_EM_) cells and tissue-resident memory T (T_RM_) cells (80). Compared with T_EM_ cells, T_RM_ cells express higher levels of IRF4, and their formation and maintenance are IRF4 dependent (32). IRF4 deletion leads to an increase in T_EM_ cells and a decrease in T_RM_ cells, but it does not affect the total number of memory T cells (32). Thus, targeting IRF4 may strongly reduce the number of T_RM_ cells, thus substantially weakening transplant rejection (81).

In addition, recent studies have shown that TCF1 is essential for maintaining CD8+ T_CM_ cells and serves as a positive biomarker for prolonged survival and effective responses to PD1 inhibitors in various solid tumors and hematological malignancies (82–85). Undoubtedly, high-level IRF4 is beneficial to the initial effector function, but sustained overexpression of IRF4 inhibits the expression of TCF1, which further damages the production of antigen-specific T_CM_ cells and is not conductive to the rapid effect function in recall responses (24). Collectively, accumulating studies have demonstrated that IRF4 is indispensable for robust proliferation and the effector function of memory T cells in recall responses.

### Persistently high IRF4-driven the exhaustion of CD8+ T cells and how to revert the exhaustion

**Figure 4:**
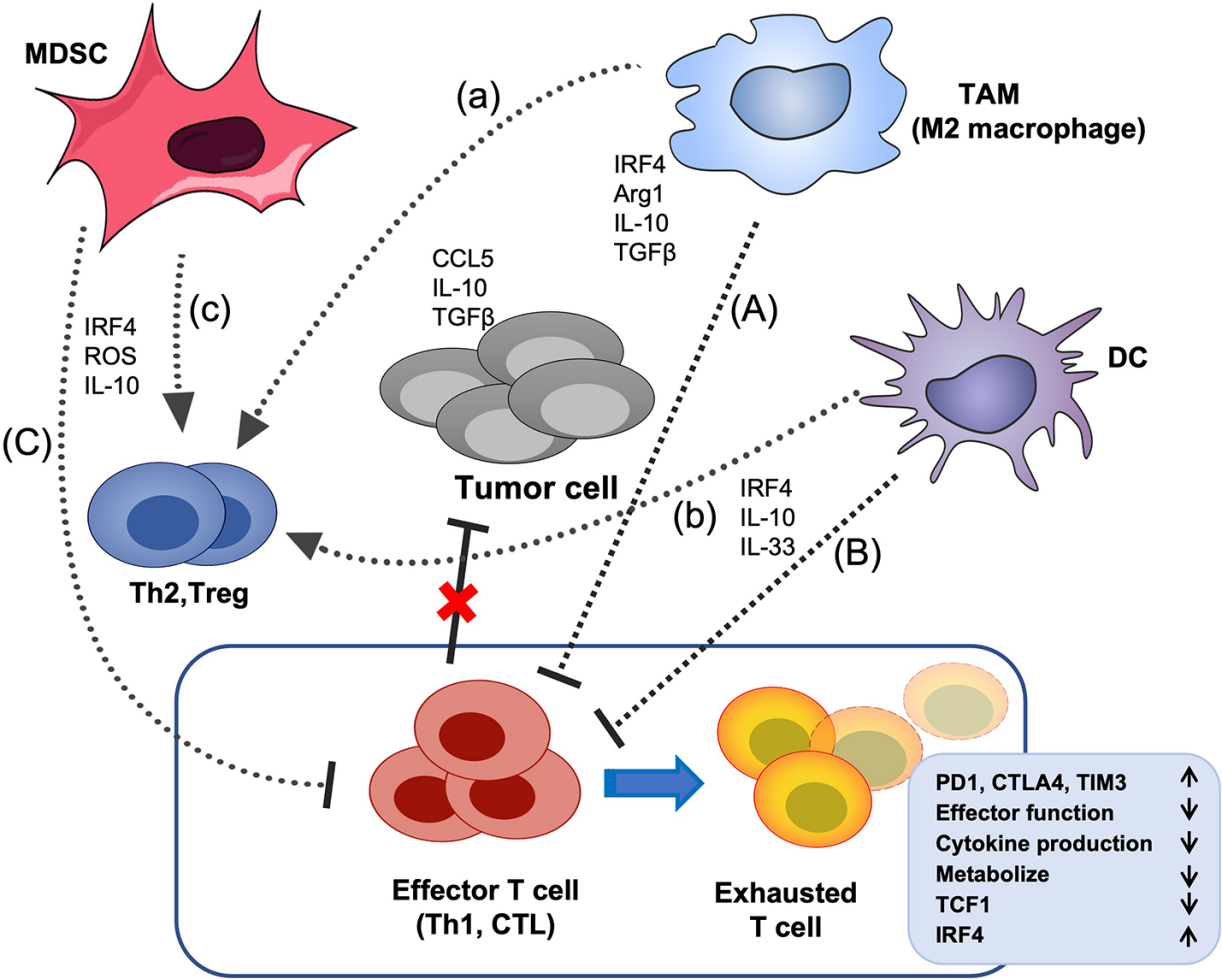
The effects of IRF4 on the crosstalk between immunosuppressive cells and T cells in the TME. Tumor cells and multiple soluble chemokines and cytokines recruit and induced various immunosuppressive cells, such as MDSCs, TAMs and DCs to the TME, which further aggravate the TME disorder and promote tumor growth. These myeloid derived immunosuppressive cells can suppress the effector function of CTL and Th1 cells and promote the differentiation of Treg cells and Th2 cells. In addition, tumor-related antigens stimulate the abnormal activation of effector T cells, ultimately, lead to the CD8+ T cell exhaustion, characterized by up-regulation of multiple inhibitory receptors, progressive loss of effector function and impaired differentiation of memory T cells, etc. IRF4 plays critical roles in the generation of various immunosuppressive cells, and the above crosstalk between myeloid derived immunosuppressive cells and effector T cells **(A–C)** and Treg cells **(a-c)** in the TME. The black arrow indicates promotion, the black horizontal line indicates inhibition, and the red cross indicates that the anti-tumor activity of effector T cells is impaired.

High IRF4 is essential for maintaining the differentiation and expansion of effector CD8+ T cells (68, 72). However, too much is as bad as too little. Persistent antigen stimulation due to tumor or chronic viral infection can cause constitutively high expression of IRF4, which in turn induces CD8+ T cell exhaustion (24). There are several characteristics of exhausted CD8+ T cells (**Figures 3A**, **4**): (1) up-regulation of multiple inhibitory receptors (86), (2) progressive loss of effector function and impaired differentiation of potential memory T cells (85, 87), (3) decreased production of cytokines involved in chemotaxis, adhesion and migration, and (4) metabolic deficiency (88). Thus, functional exhaustion is probably due to both active suppression and passive defects in signaling and metabolism.

Studies have demonstrated that the epigenetic and transcriptional programs driving CD8+ T cell exhaustion are triggered by sustained antigen-dependent activation of TCR signaling, leading to two events: (1) the sustained overexpression of TCR-responsive IRF4 and its binding partners, mainly AP1 family members, including BATF, BATF3, JunB and JunD (24, 35, 89–92), as well as nuclear factor of activated T cells (NFAT), a key regulator of T cell activation (93), followed by (2) sustained expression of multiple exhaustion-related molecules (24). Specifically, overexpressed IRF4 binding with AP1 family members or NFAT leads to an overabundance of IRF4/AP1 complexes or NFAT homodimers that are recruited to specific DNA sites to open multiple exhaustion-related chromatin regions, including inhibitory receptors, such as PD1, T-cell immunoglobulin and mucin domain 3 (TIM3) and cytotoxic T lymphocyte antigen 4 (CTLA4) (24, 35, 94, 95), as well as orphan nuclear receptor 4A (NR4A) and thymocyte selection-associated high mobility group box (TOX) family members, which act to impose exhaustion (96, 97), further inhibiting TCF1 expression (**Figure 3C**) (24, 35). These events eventually drive CD8+ T cell exhaustion and limit the development of TCF1+ memory-like T cells and anti-tumor activity (**Figure 3A**). This chromatin binding imbalance due to the accumulation of IRF4/AP1 TF complexes was also found in CAR-T cell therapy (89).

Fortunately, Lynn et al. (89) found that ectopic overexpression of c-Jun in exhausted CAR-T cells can effectively rescue exhaustion and restore anti-tumor activity by disrupting and/or displacing immunosuppressive transcriptional complexes containing IRF4 and AP1 family members (89). Moreover, based on the overexpression of BATF and IRF4 in exhausted T cells (89, 98), knockdown of BATF or IRF4 could remarkably enhance the tumor-killing ability of CAR-T cells by reversing their exhaustion and prolonging their persistence (89, 90). Likewise, Seo et al. (25) found that overexpressed BATF in BATF-transduced CAR-T cells could cooperate with appropriate amount of IRF4 to counteract exhaustion, promoting the expansion of CD8+ CAR-T cells and increasing their effector cytokine production. Nevertheless, inhibiting the interaction between BATF and IRF4 will greatly weaken the tumor control ability of BATF-overexpressing CAR-T cells (25).

Collectively, these findings show that persistent overexpression of IRF4 drives T cell exhaustion depending on the specific microenvironment and the amount and functional status of its binding partners. Therefore, manipulating the formation of IRF4/AP1 complexes may be an inspiring therapeutic strategy to overcome T cell exhaustion. Yet, the core transcriptional network of IRF4 involved in these two opposing programs still needs to be further elucidated.

## Regulation of IRF4 in immunosuppressive cells in the TME

Various immunosuppressive cells and multiple soluble chemokines and cytokines in the TME interact to not only establish an immunosuppressive TME but also directly or indirectly inhibit the proliferation and activation of CD8+ T cells (99, 100), which may cause chemoresistance and failure of immunotherapy and facilitate tumor growth and metastasis (101–103). IRF4 plays important and complicated roles in the development and function of immunosuppressive cells and their interaction with T cells (**Figure 4**) (104, 105).

### IRF4 promotes the polarization of M2 macrophages in the TME

There are two types of macrophages: M1 (anti-tumor activity) (106, 107) and M2 (pro-tumor activity) (108). Generally, TAMs mainly refer to M2 macrophages, which are characterized by high expression of arginase1 (Arg1), chitinase-like 3 (Ym1/Chil3), found in inflammatory zone 1 (Fizz1) and mannose receptor (MR) (109, 110). IRFs play a key role in macrophage maturation and phenotypic polarization. Of the nine IRFs, IRF1, IRF5 and IRF8 are involved in the commitment of M1 macrophages, whereas IRF3 and IRF4 are crucial for M2 macrophage polarization through regulating the expression of Arg1 and Ym1, which further sufficiently produces Th2 and directly suppresses effector T cell proliferation (111–113).

In addition, it has been reported that Jumonji domain containing 3 (Jmjd3) is essential for M2 macrophage polarization, and IRF4 is a Jmjd3 target gene (110, 114). Phosphatidylserine released by apoptotic tumor cells could induce the polarization and accumulation of M2 macrophages *via* a STAT3-Jmjd3-IRF4 signaling axis (115); therefore, down-regulation of Jmjd3 by targeting the STAT3-Jmjd3-IRF4 axis may be a candidate approach for inhibiting the accumulation of M2 macrophages in tumor sites and remodeling the TME. Moreover, some miRNAs have been found to promote the transformation of macrophages from M2 to M1 by targeting IRF4 to activate IRF5 (116, 117). Given that IRF4 promotes the polarization of M2 macrophages, targeting IRF4 to reprogram TAM polarization in the TME appears to be a promising therapy for tumors.

### IRF4 is beneficial to DC differentiation in the TME

DCs, known as professional antigen presenting cells, play a major role in orchestrating immune responses, and can be mainly divided into three subtypes: plasmacytoid DCs (pDCs), classical DCs (cDCs, including cDC1 and cDC2), and monocyte-derived DCs (mo-DCs) (9, 118, 119). However, the differentiation and maturation of DCs are often impaired by the immunosuppressive TME, which leads to DC dysfunction and induces tolerance to tumor cells (8, 9, 118, 119). For instance, mature pDCs exert immunostimulatory function, which is characterized by the production of large amounts of type I IFNs. Whereas, in the TME, pDCs with reduced production of type I IFNs favor the development of Treg cell, exert immunosuppressive effects on CTLs and promote tumor progression (8, 9, 120, 121). Several studies have indicated a role for IRF4 in development of monocytes, pDCs, and cDCs (122–124). IRF4 contributes to the differentiation of pDCs (122). In addition, IRF4 plays a key role in the development of cDC2 and promotes their survival and migration to lymph nodes and is essential TF for cDC2-mediated Th2 induction (122). By contrast, inhibition of IRF4 in DCs represses Th2 and promotes Th17 responses (123).

The monocytes in the TME can prioritize differentiation into monocyte-derived macrophages (mo-Macs) rather than mo-DCs (3, 105). The presence of mo-DCs has been correlated with CD8+ T cell activation and successful anti-tumor therapy (125). IRF4 is essential for human mo-DC differentiation and efficient antigen cross-presentation, whereas IRF4-deficent monocytes are phone to differentiation into mo-Macs (124). Devalaraja et al. found that the TME induces tumor cells to produce retinoic acid (RA) in murine sarcoma models, which drives intratumor monocyte polarization to mo-Macs instead of mo-DCs by inhibiting IRF4 (3). Interestingly, overexpression of IRF4 in human monocytes can sufficiently block RA-mediated mo-Mac differentiation (3, 124). Collectively, these results suggest that IRF4 plays critical and complicated roles in the maturation and differentiation of DCs in the TME.

### Tumor and MDSC-restricted IRF4 expression enhances the suppressive activity of MDSCs and promotes the immunosuppressive TME

MDSCs are immature myeloid cells that do not differentiate into mature myeloid cells, and this is a major obstacle to achieving successful immunotherapy in tumors (126, 127). Two major subpopulations, monocytic (M) MDSCs and polymorphonuclear (PMN)-MDSCs, have an immune suppressive function. IRF4 plays a role in the lymphoid cell development. However, IRF4 expression is decreased in immature myeloid cells, such as MDSCs in tumor-bearing mice and chronic myeloid leukemia cells (104, 128). Accordingly, IRF4 deficiency further favors the generation of MDSCs in the TME, and increases the expansion of M-MDSCs and the infiltration of PMN-MDSCs with a strong suppressive capacity, which inhibits the proliferation of CD8+ T cells through IL10 and ROS generation and promotes tumor growth (104, 129). By contrast, an increase in the IRF4 expression in MDSCs from bone marrow cells inhibits the numbers of MDSCs through induction differentiation, and further damages the immunosuppressive function of MDSCs (104). Unfortunately, IRF4 expression is remarkably suppressed during the development of MDSCs and tumor formation in the TME (104).

Altogether, these data show that IRF4 plays a critical role in preventing the generation of MDSCs; nevertheless, IRF4 expression is limited by tumors and MDSCs, which may in turn boost the accumulation and suppressive activity of MDSCs to accelerate the generation of an immunosuppressive TME. Thus far, the exact mechanisms regulating IRF4 in the differentiation of MDSCs remains largely unknown.

## Conclusion and future prospects

IRF4 plays key roles in the development of various immunosuppressive cells in the TME. More importantly, this TF is also indispensable in the differentiation and function of effector T cells, particularly memory T cells in the secondary response (32, 64, 78). Notably, the amount and duration of IRF4 expression determines CD8+ T cell differentiation into effector T cells or exhausted T cells, depending on the specific microenvironment and states of its binding partners (24, 34, 35, 91). Thus far, the dual regulatory mechanism of IRF4 in T cell immunity is not completely clear. Given the imbalance between the activating and immunoregulatory IRF4/AP1 complexes induced by persistent high expression of IRF4 and AP1 family members in specific contexts, manipulating the composition of the IRF4/AP1 complexes may be a novel therapeutic strategy for overcoming T cell exhaustion and improving anti-tumor potency.

Recently, several studies have reported exciting findings, including the regulation of the physical interaction between IRF4 and its binding partners, the formation of ternary complexes through overexpression of BATF or c-Jun, and the regulation of the amount of IRF4 or BATF, which are essential for rescuing exhaustion and improving anti-tumor potency in tumor-specific CAR-T cells (25, 89, 90). In addition, several recent studies have focused on targeting Roquin and Regnase1, negative regulators of T cell activation and differentiation, to enhance the proliferation and persistence of tumor-antigen-specific CD8+ T cells or CAR-T cells and effectively inhibit tumor growth (71, 130–132). In fact, the beneficial effects of the regulation of these targets are caused not only by loss of function of a single gene, but likely also caused by the cooperative regulation of multiple targets. For instance, the promotion of the survival and proliferation of tumor-antigen-specific CD8+ T cells by inactivating Roquin1 is highly dependent on the expression of IRF4 (71). Similarly, Regnase1 deficiency contributed to CAR-T cell survival and proliferation, which also specifically required BATF (130), further enhancing recall responses by increasing TCF1+ CAR-T cell population (131). By coincidence, proper reduction of IRF4 contributes to the generation of TCF1+ memory T cells that control tumor recurrence (25). Together, these findings point to promising new targets for improving immunotherapy.

Taken together, based on the close cooperation and regulatory relationships between IRF4, BATF, TCF1 and Roquin or Regnase1, targeting IRF4 or IRF4-based multi-target combination is an important direction for regulating human anti-tumor T cell immunity and the TME to improve therapeutic efficacy in the future.

## Author contributions

JL reviewed the literature and wrote the manuscript. TL and PL contributed to literature collection and manuscript revision. QY designed the review, and wrote and revised the manuscript. All authors contributed to the article and approved the submitted version.
